# Cytoplasmic aryl hydrocarbon receptor regulates glycogen synthase kinase 3 beta, accelerates vimentin degradation, and suppresses epithelial–mesenchymal transition in non-small cell lung cancer cells

**DOI:** 10.1007/s00204-016-1870-0

**Published:** 2016-10-17

**Authors:** Ching-Hao Li, Chen-Wei Liu, Chi-Hao Tsai, Yi-Jen Peng, Yu-Hsuan Yang, Po-Lin Liao, Chen-Chen Lee, Yu-Wen Cheng, Jaw-Jou Kang

**Affiliations:** 1 Department of Physiology, School of Medicine, College of Medicine, Taipei Medical University, Taipei, Taiwan; 2 Graduate Institute of Medical Sciences, College of Medicine, Taipei Medical University, Taipei, Taiwan; 3 School of Pharmacy, Taipei Medicine University, 250 Wu-Hsing Street, Taipei, Taiwan; 4 Institute of Toxicology, College of Medicine, National Taiwan University, 1 Jen-Ai Road, Section 1, Taipei 10, Taiwan; 5 Department of Pathology, Tri-Service General Hospital, National Defense Medical Center, Taipei, Taiwan; 6 Department of Microbiology and Immunology, School of Medicine, China Medicine University, Taichung, Taiwan

**Keywords:** Aryl hydrocarbon receptor, Epithelial–mesenchymal transition, Vimentin, Glycogen synthase kinase 3 beta, Ubiquitin

## Abstract

Aryl hydrocarbon receptor (AHR), a ligand-activated transcription factor, has been studied extensively in carcinogenesis through the genomic pathway. In recent years, AHR has also been reported to exert positive or negative effects on epithelial–mesenchymal transition (EMT), the crucial step in tumor malignant progression. However, the detailed mechanism remains controversial. Analysis of AHR-expression levels in non-small cell lung cancer cell lines and lung cancer tissues revealed an inverse correlation between AHR protein levels and tumor cell invasion and metastasis. Overexpression of wild-type AHR in H1299 cells (AHR poorly expressed, potently invasive) not only accelerated mesenchymal vimentin degradation, but also prevented cell invasion in vitro and in vivo. In the absence of AHR agonists, the overexpressed AHR protein was predominantly localized in the cytoplasm, where it interacted with vimentin and functioned as an E3 ubiquitin ligase. A 6-h incubation with the proteasome inhibitor MG-132 fully rescued vimentin from AHR-mediated proteasomal degradation. In AHR-overexpressing H1299 cells, either vimentin degradation or invasive suppression could be reversed when glycogen synthase kinase 3 beta (GSK3β) was inactivated by CHIR-99021 treatment. In contrast, silencing of AHR in A549 cells (AHR highly expressed, weakly invasive) resulted in the downregulation of epithelial biomarkers (E-cadherin and claudin-1), augmentation of mesenchymal vimentin level, and GSK3β Ser-9 hyper-phosphorylation, which led to enhanced invasiveness. This work demonstrates that cytoplasmic, resting AHR protein may act as an EMT suppressor via a non-genomic pathway. Depletion of cytoplasmic AHR content represents a potential switch for EMT, thereby leading to the scattering of tumor cells.

**Electronic supplementary material:**

The online version of this article (doi:10.1007/s00204-016-1870-0) contains supplementary material, which is available to authorized users.

## Introduction

Lung cancer, predominantly non-small cell lung carcinoma (NSCLC), is an aggressive malignant disease with high morbidity and mortality in the world. Although a number of treatment options are improving continuously, unfortunately, the prognoses are still unfavorable. The poor prognoses are highly associated with the acquired resistance of tumor against chemotherapy or targeted therapies. Among the various proposed mechanisms for chemoresistance in tumor cells, metastatic behavior has been reported to be critical (Atmaca et al. 2015).

Epithelial–mesenchymal transition (EMT) is a fundamental process that governs embryonic development. This transition is characterized by profound phenotypic disruption in intercellular adhesion. In most cases, this is mediated by SNAI2/SLUG-dependent transcriptional repression of epithelial signatures (e.g., E-cadherin). In parallel, migratory and invasive properties are gained by enhanced expression of mesenchymal factors such as α-smooth muscle actin, vimentin, fibronectin, and matrix metalloproteinases (MMPs). To date, it is believed that EMT processes are involved during the malignant conversion of epithelial tumors (Rico-Leo et al. 2013). During EMT, cancer cells not only de-differentiated to fibroblastoid, stem cell-like phenotypes with high motility, and invasive properties, but also increased production of extracellular matrix to modify tumor microenvironment.

Aryl hydrocarbon receptor (AHR) is a ligand-activated transcriptional regulator, a member of the basic helix-loop-helix/Per-Arnt-Sim (bHLH/PAS) family. It is ubiquitously expressed in multiple organs and tissues. The PAS region responds to its ligands, many of which are environmental toxins, such as 2,3,7,8-tetrachlorodibenzo-p-dioxin (TCDD), halogenated aromatic hydrocarbons (HAHs), and benzo(a)pyrene (B[a]P). In the absence of ligands, AHR is sequestered in the cytosol by forming a hetero-complex with heat-shock protein 90 (Hsp90) through both the bHLH and PAS regions. Upon ligand binding, AHR rapidly escapes from its chaperones and translocates into the nucleus, where it dimerizes with AHR nuclear translocator (ARNT) and recruits a set of coactivators and/or corepressors. Eventually, a battery of AHR-target genes with xenobiotic-responsive elements (XREs, with the core sequences 5′-T/GCGTG-3′) in their promoter region is induced. Most of them are xenobiotic-metabolizing enzymes (e.g., cytochrome P450 1A1), which are involved in the biotransformation or detoxification of harmful xenobiotics (Fan et al. 2010). Further, the metabolism of many pro-carcinogens to active intermediates via an AHR-dependent genomic pathway that results in DNA adduct formation, mutagenesis, and tumorigenesis has been well characterized (Ikuta et al. 2009; Safe et al. 2013).

Abnormal expression or activity of AHR has been highlighted in malignant tumor progression in the past few years. AHR immunoreactivity analysis of tissues from cancer patients revealed increased expression levels of AHR in breast (Saito et al. 2014), esophageal (Zhang et al. 2012), gastric (Peng et al. 2009b), pancreatic (Koliopanos et al. 2002), prostate (Richmond et al. 2014), lung (Su et al. 2013; Portal-Nuñez et al. 2012), liver (Liu et al. 2013), and urothelial carcinoma (Ishida et al. 2010) as well as glioblastomas (Gramatzki et al. 2009), as compared to its expression in surrounding normal tissues. In most cases, high AHR expression is found in the nucleus and significantly correlates with the histological grade. However, in esophageal, pancreatic, and breast tumors, AHR is localized in the cytoplasm and the AHR-expressing status is inversely correlated with the histological grade of patients. The complex pattern of AHR expression and localization in tissues with different malignancies raised our interest to investigate the function of AHR in EMT regulation.

It has been previously reported that exposure to AHR agonists such as TCDD results in the upregulation of snail, slug, and mesenchymal biomarkers (e.g., MMP-2, MMP-9, fibronectin, and vimentin), whereas epithelial biomarkers (e.g., claudin-1 and E-cadherin) are downregulated (Peng et al. 2009a; Ishida et al. 2010; Belguise et al. 2007). These changes are not observed in AHR-KO mice or by pretreatment with AHR-specific siRNA (Pierre et al. 2014), suggesting that the transactivation of AHR is necessary for EMT. Besides the genomic pathway described above, ligand binding also activates non-genomic pathways (defined as AHR dependent, but ARNT independent), which lead to Src kinase activation and cell migration (Randi et al. 2008; Tomkiewicz et al. 2013). However, whether the cytoplasmic, resting (unliganded) form of AHR regulates EMT has never been studied. Toward this end, we engineered a NSCLC cell line H1299 (AHR deficient, potently invasive) to express AHR constitutively. Subsequently, we detected the overexpressed AHR protein in the cytosol, where the AHR–vimentin complex was formed, leading to vimentin degradation via the proteasome pathway. In contrast, when AHR expression was knocked-down in the A549 cell (AHR highly expressed, weakly invasive), transformation to a more aggressive phenotype was observed. Thus, we concluded that unliganded AHR in the cytoplasm may negatively regulate EMT.

## Materials and methods

### Cell culture, plasmids, and transfection

Human NSCLC cell lines, A549 and H1299, were purchased from American Type Culture Collection (ATCC; Manassas, VA) and were cultured in Dulbecco’s modified Eagle’s medium (DMEM) as described previously (Liu et al. 2014). The CL1-0 and CL1-5 cells are sublines, derived from human lung adenocarcinoma tissues, according to the differences in metastatic potential. Both sublines were gifts from Dr. Huei-Wen Chen (National Taiwan University, Taipei, Taiwan) and were maintained in RPMI-1640 media supplemented with 10 % FBS (Liu et al. 2014).

The plasmid expressing wild-type hAHR was constructed by using pcDNA™ 3.1/V5-His TOPO^®^ TA expression kit (Invitrogen, Carlsbad, CA). The insertion sequence of hAHR (Gene ID: 196; NCBI reference sequence: NM_001621.4) was obtained by PCR with the primer set (sense: ATGAACAGCAGCAGCGCCAAC; antisense: TTACAGGAATCCACTGGATGT). For transient transfection, cells were seeded at a density of 5 × 10^5^ cells/well. After an overnight incubation, pcDNA3.1 or pcDNA3.1-AHR was introduced into cells by using Turbofect transfection reagent and manufacturer’s recommended protocol (Thermo Scientific™).

For hAHR silencing, the pLKO.1-puro vector (TRCN0000021254 and TRCN0000021258), which expressed a small hairpin RNA (shRNA) specific to hAHR sequences CCCACAACAATATAATGTCTT and CGGCATAGAGACCGACTTAAT, respectively, was purchased from RNAi core of Academia Sinica (Taipei, Taiwan). The stabilized AHR-silenced A549 clone was obtained after a 2-week puromycin (1 μg/mL) selection.

### Cell invasion assay

Twenty-four hours after transfection, cells were detached from culture plates. Subsequently, 0.2 mL of the cell suspension (5 × 10^5^ cells/mL) was added to the upper chamber of transwell with 8-μm pore size (Corning, New York, NY, USA). Transwell was precoated with 0.1 mL diluted Matrigel™ (1:4 dilution with serum-free DMEM) (BD Biosciences, Belford, MA). Then, culture medium was added to the lower chamber. After 24-h incubation at 37 °C, cells were fixed with 3.7 % formaldehyde and stained in 0.1 % crystal violet for 15 min, and non-migratory cells were removed by scraping the upper surface of the chamber membrane. The invasive cells were counted under a light microscope (Ishida et al. 2015).

### MTT colorimetric assay

The growth curves of distinct cells were determined by the effective reduction of the tetrazolium dye, 3-(4,5-dimethylthiazol-2-yl)-2,5-diphenyltetrazolium bromide (MTT) to formazan (Tsai et al. 2015).

### Western blot analysis

Whole-cell extracts were prepared by scraping in 0.2–0.3 mL of RIPA lysis buffer as described earlier (Tsai et al. 2015). Nuclear and cytoplasmic fractions were harvested by using the Nuclear Extract Kit and following the manufacturer’s instructions (Active Motif, Carlsbad, CA). Protein concentrations were determined by Bradford method (BioRad, Hercules, CA). Equal amounts of protein from each treatment were diluted with loading buffer, boiled, and separated on 10 or 15 % gels by sodium dodecyl sulfate-polyacrylamide gel electrophoresis (SDS-PAGE). Then, the separated proteins were transferred to polyvinylidene difluoride (PVDF) membranes. After a short blocking with non-fat milk, the membranes were probed with appropriate primary antibodies, including AHR, ARNT (Santa Cruz), vimentin, β-catenin, N-cadherin, E-cadherin, claudin-1 (Genetex), occludin (Proteintech), ZO-1 (Invitrogen), lamin A/C, GSK3β phospho-Ser-9 and phospho-Tyr-216, GSK3β (Epitomics), and β-actin (Sigma), followed by an incubation with horseradish peroxidase-conjugated secondary antibodies. Immunocomplexes were visualized using the enhanced chemiluminescence (ECL) detection reagent (Millipore). Band intensities were determined by densitometry analysis using Gel-Pro Analyzer software (Media Cybernetics, Warrendale, PA).

### Reverse transcription polymerase chain reaction

Total RNA samples (3 μg), which extracted with a commercial kit from Roche (Germany), were converted into complementary DNA sequences (cDNA) by MMLV SuperScript II transcriptase (Epicentre, Madison, WI). Then, resultant cDNA was used as a template in a PCR using Taq polymerase mixture. The following amplification conditions were used: an initial denaturation at 95 °C for 5 min, followed by 25–30 cycles of denaturation at 95 °C for 30 s, annealing at 60 °C for 30 s, and extension at 72 °C for 1 min, followed by a final extension at 72 °C for 10 min. PCR products were resolved on a 2 % agarose gel and visualized by ethidium bromide staining. Band intensities were determined by densitometry analysis in Gel-Pro Analyzer software (Media Cybernetics, Warrendale, PA). β-Actin or GAPDH mRNA was used as a normalization control. The forward and reverse primers used in this study are presented in supplementary Table 1.

### Immunofluorescence staining

Cells were grown on glass coverslips to 70 % confluence. After treatment, samples were washed twice with PBS and fixed in 4 % paraformaldehyde for 15 min. Then, cells were permeabilized with 0.1 % Triton X-100 for 10 min, followed by 10-min incubation with 5 % horse serum for blocking non-specific epitopes. AHR localization was detected with an anti-AHR antibody (Santa Cruz, sc-74571), whereas normal mouse IgG was used as control antibody. After an overnight incubation at 4 °C, samples were washed routinely with PBS and then incubated with fluorescein-conjugated secondary antibody at room temperature for 1 h. 4′,6-Diamidino-2-phenylindole (DAPI) was used for nuclear staining. Finally, samples were mounted and images were taken using a Zeiss fluorescence microscope (Carl Zeiss, Jena, Germany).

### Luciferase reporter assay

Cells were transfected with 2 μg pGL4.43[luc2P/XRE/Hygro] vector (Promega, Madison, WI) and 0.5 μg of pRK5-LacZ, as described earlier. After 24-h transfection, cells were exposed to B(a)P for 6–12 h. Subsequently, cell extracts were harvested. The luciferase activity was measured using the Promega reporter assay system. Luciferase activities were normalized to transfection efficiency using the pRK5-LacZ values.

### Immunoprecipitation

Whole-cell lysates were precleared by a short incubation with protein A magnetic beads (Millipore). Then, the lysates (1 mg) were subjected to immunoprecipitation by the addition of 1 μg/mL of the following captured antibodies: anti-AHR (Santa Cruz, sc-74571) or antivimentin (Genetex, GTX100619) and incubated overnight with end-on-end rotation at 4 °C. This was followed by shaking with 50-μL protein A magnetic beads for another 2 h. The captured immunocomplexes were precipitated and washed 3 times with RIPA buffer prior to the addition of 100 μL of 2 × SDS sample buffer and heating at 95 °C for 5 min. Samples were separated by 10 % SDS-PAGE and analyzed as described earlier (Li et al. 2014).

### Lung colonization assay

C57BL/6 J mice (4–6 week old, male) were obtained from National Taiwan University Animal Center and housed aseptically in the animal facility. Mice had free access to water and rodent chow. All experimental procedures were performed in accordance with the Guide for the Care and Use of Laboratory Animals and were approved by the IACUC. The metastatic potential evaluation in vivo was conducted as described previously (Liu et al. 2014). Briefly, single-cell suspensions prepared in serum-free DMEM (2.5 × 10^5^ cells/mL) were injected into the tail vein of C57BL/6 J mice (0.2 mL/mice). Thirty days later, the mice were anesthetized with isoflurane and killed. The lungs were fixed with Bouin’s solution, and metastatic colonies on the lung surface were counted macroscopically.

### Immunohistochemical (IHC) analysis

Human lung cancer tissue microarray (CCA4 E65) was purchased from Pantomics (Richmond, CA). The slides were stained for AHR at the Department of Pathology, National Taiwan University Hospital, according to their standard operation protocol. The tissue specimen grades, as well as the immunoreactive score, were confirmed by an experienced pathologist (Dr. Yi-Jen Peng, Department of Pathology, Tri-Service General Hospital, National Defense Medical Center, Taipei, Taiwan).

### Statistical analysis

All data are expressed as mean ± standard deviation (*N* ≥ 3). One-way analysis of variance (ANOVA) was adopted for statistical analysis among groups. The statistical differences between the control and individual testing conditions were analyzed by Student’s *t* test. *p* values of <0.05 were considered statistically significant.

## Results

### A low AHR-expression level in human lung cancer tissues/cells is significantly associated with its malignancy

Correlation between AHR expression and tumor clinicopathological features was analyzed in lung cancer tissues of 26 male and 4 female patients, the results of which are summarized in Table 1. Representative images of AHR immunohistochemistry staining are shown in supplementary Fig. 1. While no statistically significant correlation was found between AHR expression and parameters related to age (*p* = 0.366), gender (*p* = 0.661), histological grade (*p* = 0.878), and regional lymph nodes metastasis (*p* = 0.88), the metastatic status of tissues showed a significant correlation (*p* = 0.018) with AHR-expression levels.

**Table 1:** Relationships between AHR expression and clinicopathological parameters of lung cancer

	*n*	AHR expression		
		Positive (%)	Negative (%)	*p* value
Total	30	18 (60)	12 (40)	
Age				
>60	13	9 (70)	4 (30)	0.366
≤60	17	9 (53)	8 (47)	
Gender				
Male	26	16 (62)	10 (38)	0.661
Female	4	2 (50)	2 (50)	
Cell type				
Adenocarcinoma	7	4 (57)	3 (43)	0.064
Squamous cell carcinoma	12	9 (75)	3 (25)	
Adenosquamous carcinoma	3	3 (100)	0 (0)	
Metastatic adenocarcinoma	8	2 (25)	6 (75)	
Grade				0.878
1	6	4 (67)	2 (33)	
2	11	6 (55)	5 (45)	
3	13	8 (62)	5 (38)	
TNM				
T1	1	0 (0)	1 (100)	0.09
T2	19	14 (74)	5 (26)	
T3	10	4 (40)	6 (60)	
N0	9	5 (56)	4 (44)	0.88
N1	9	6 (67)	3 (33)	
N2	12	7 (58)	5 (42)	
M0	22	16 (73)	6 (27)	0.018
M1	8	2 (25)	6 (75)	

**Fig. 1:**
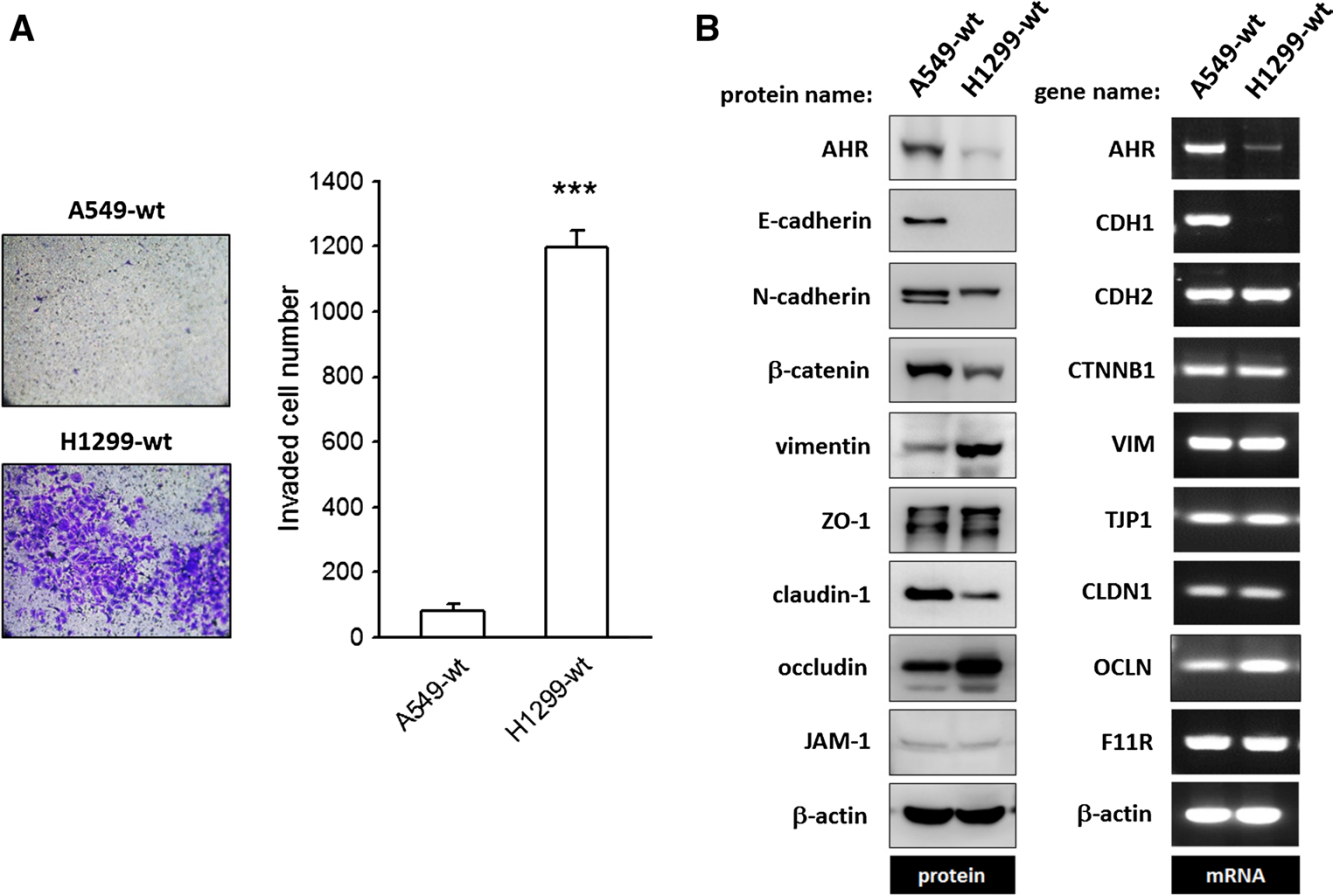
The invasive potential of NSCLC cancer cell lines, A549 and H1299, is related to the amount of AHR protein expressed. **a** The invasive ability of A549 and H1299 was evaluated by using Matrigel-precoated transwells as described in Materials and Methods. Both representative images and quantitative data demonstrated H1299 is more aggressive than A549. ***p < 0.001 indicates a statistically significant difference from A549. **b** Representative images of immunoblot and agarose gel electrophoresis of PCR products to detect and compare protein and mRNA expression of epithelial–mesenchymal biomarkers between A549 and H1299 cells. At the mRNA level, besides AHR and E-cadherin, no differences were found between A549 and H1299. An inverse relationship was found between AHR protein expression level and vimentin. For HGNC name/symbol of genes and encoded proteins, please refer to Supplementary Table 1

The invasive potential of human NSCLC cell lines was evaluated using Matrigel-precoated transwell inserts. The significantly higher counts of invaded cells obtained for H1299 as compared to A549 cells reflect the potent invasive ability of these cells (Fig. 1a). The mRNA and protein expression levels of AHR and epithelial–mesenchymal biomarkers were analyzed, and the representative images are presented in Fig. 1b. In accordance with its strong invasive potential, H1299 cells express high protein levels of vimentin, but not of β-catenin, claudin-1, or AHR, as compared to the weakly invasive A549 cell line. E-cadherin, the prototypical marker of epithelial cells, is largely expressed in A549, but not in H1299 cells, while ZO-1 and JAM-1 protein levels were identical in H1299 and A549 cells. On the other hand, comparison of mRNA expression levels in H1299 and A549 cells revealed no significant differences in transcript levels of the aforementioned EMT markers except for E-cadherin and AHR. To further corroborate the above observations, we carried out mRNA and protein expression analysis of EMT biomarkers in two other cell lines, namely: CL1-0 and CL1-5, which are subclones isolated from a 64-year-old man with lung adenocarcinoma (Chu et al. 1997). CL1-5, which is more aggressive in transwell invasion assay than precancerous CL1-0 subclone, is E-cadherin deficient and instead expresses N-cadherin, while AHR expression is negligible. In contrast, vimentin is highly expressed in CL1-5 cells (supplementary Fig. 2). Taken together, these results indicate that AHR and/or vimentin expression levels strongly correlate with the invasive potential of NSCLC cells. Specifically, downregulation of AHR leads to enhanced vimentin expression and EMT in NSCLC cells.

### AHR overexpression results in a dramatic reduction of the invasive potential of H1299, both in vitro and in vivo

**Fig. 2:**
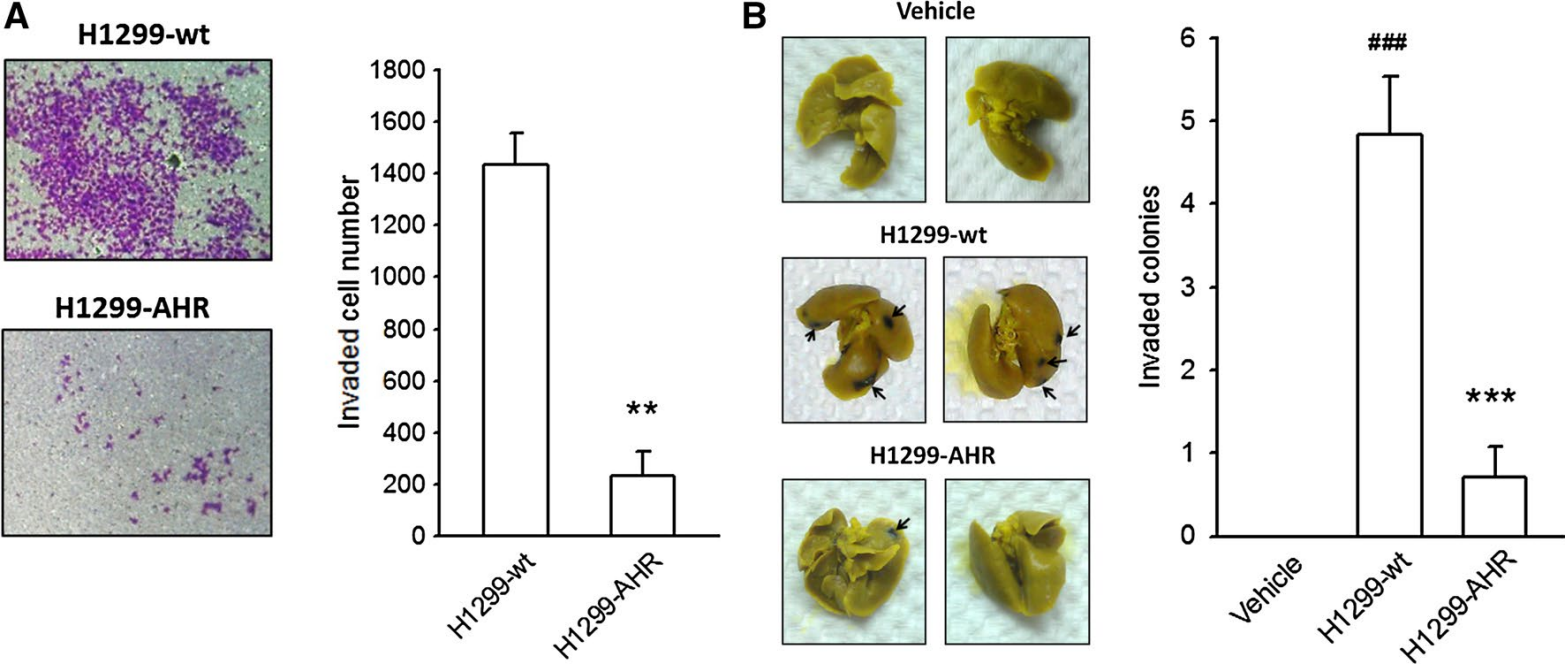
The invasive potential of H1299-AHR is reduced in vitro and in vivo. H1299-AHR (AHR-overexpressing H1299) was established as described in Materials and Methods. Representative photograph and quantitative data illustrate that the invasive potential of H1299-AHR is reduced significantly in vitro (**a**) and in vitro (**b**). **p < 0.01 and ***p < 0.001 indicate a statistically significant difference from H1299-wt. ###*p* < 0.001 indicates a statistically significant difference from vehicle control

To validate the putative tumor suppressor-like function of AHR, H1299 cells were engineered to constitutively express wild-type AHR protein (H1299-AHR). In transwell invasion assays, the number of H1299-AHR cells migrating to Matrigel was reduced significantly as compared to H1299-wt (wild type) cells (Fig. 2a). No differences in cell morphology and proliferative curve were observed between H1299-wt and H1299-AHR (supplementary Fig. 3), suggesting that the switch in invasive potential was unrelated to cell proliferation. The metastatic capacity of H1299-wt and H1299-AHR was evaluated in vivo by using lung colonization assay. As compared to vehicle-injected control, a remarkable increase in the number of metastatic nodules on the lung surface was observed in the H1299-wt-treated group, whereas a decrease in the number of metastatic colonies was seen in the H1299-AHR-treated group (Fig. 2b). Thus, both in vivo and in vitro data demonstrate that AHR overexpression can suppress the invasiveness of NSCLC cells.

### The expression of mesenchymal vimentin is reduced in AHR-overexpressing H1299 cells

**Fig. 3:**
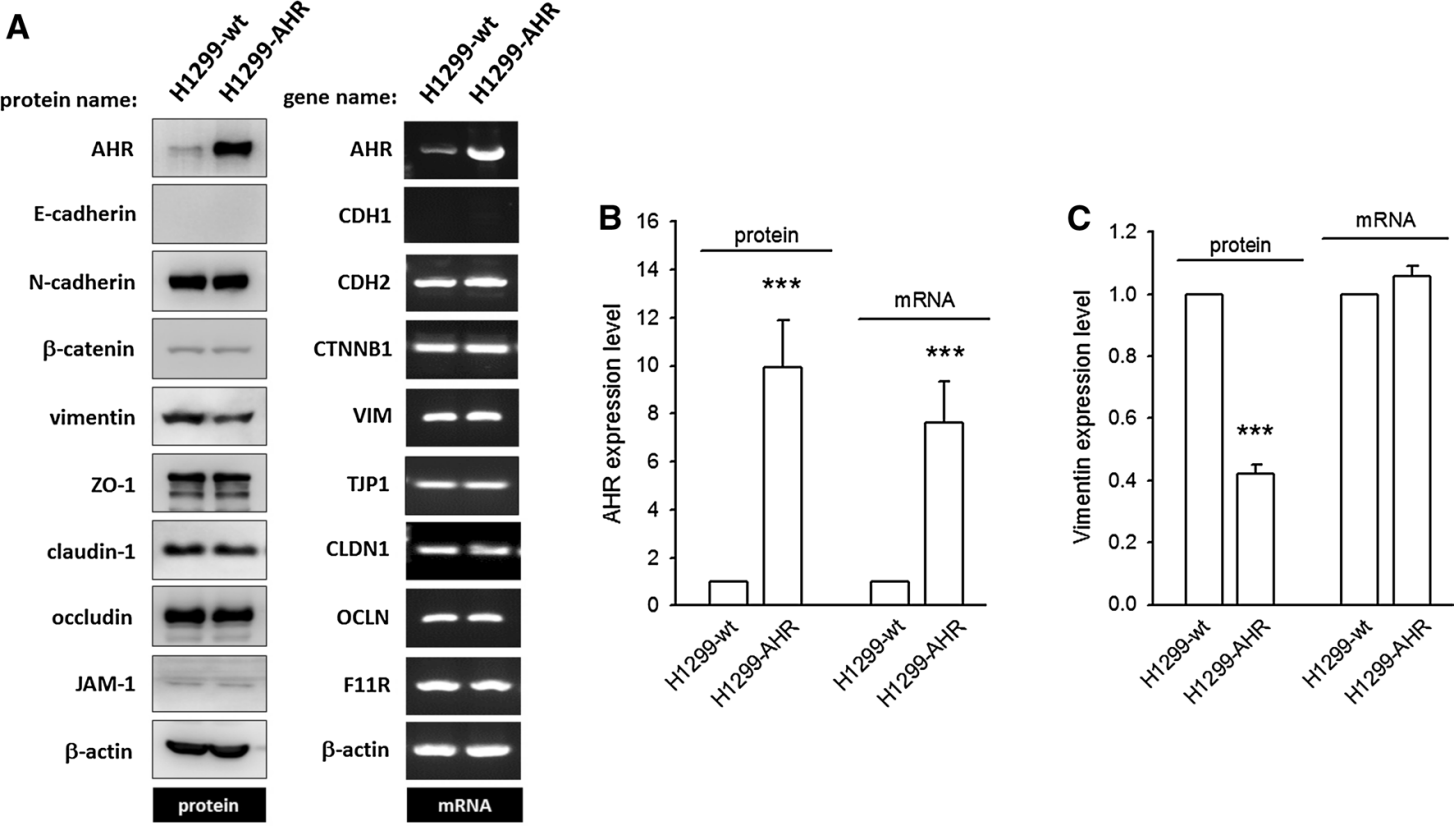
The expression of mesenchymal vimentin is reduced in H1299-AHR. **a** Representative images of immunoblot and agarose gel electrophoresis of PCR products to detect and compare protein and mRNA expression of epithelial–mesenchymal biomarkers between H1299-wt and H1299-AHR. None of the detected genes is altered at the mRNA level, with the exception of AHR, which was overexpressed by transient transfection. At the protein level, among tested factors, AHR is augmented and vimentin is reduced significantly (**b**/**c**). ***p < 0.001 indicates a statistically significant difference from H1299-wt

To determine the basis of AHR-mediated EMT suppression, the protein (or mRNA) expression levels of epithelial and mesenchymal biomarkers were examined. We found that AHR overexpression (as evidenced by a 9.92 ± 1.97- and 7.64 ± 1.71-fold increase in protein and mRNA levels, respectively) significantly downregulates vimentin protein levels (0.42 ± 0.03-fold decrease, *p* < 0.001), but not mRNA levels as compared to H1299-wt (Fig. 3; supplementary Fig. 4). The amounts of E-cadherin, N-cadherin, β-catenin, occludin, ZO-1, JAM-1, and claudin-1 are not altered on AHR overexpression, either at the protein or at mRNA level. These data indicate that AHR overexpression can repress cell invasion through translational or post-translational downregulation of vimentin.

### Overexpressed AHR protein predominantly localizes in the cytosol, rather than the nucleus

**Fig. 4:**
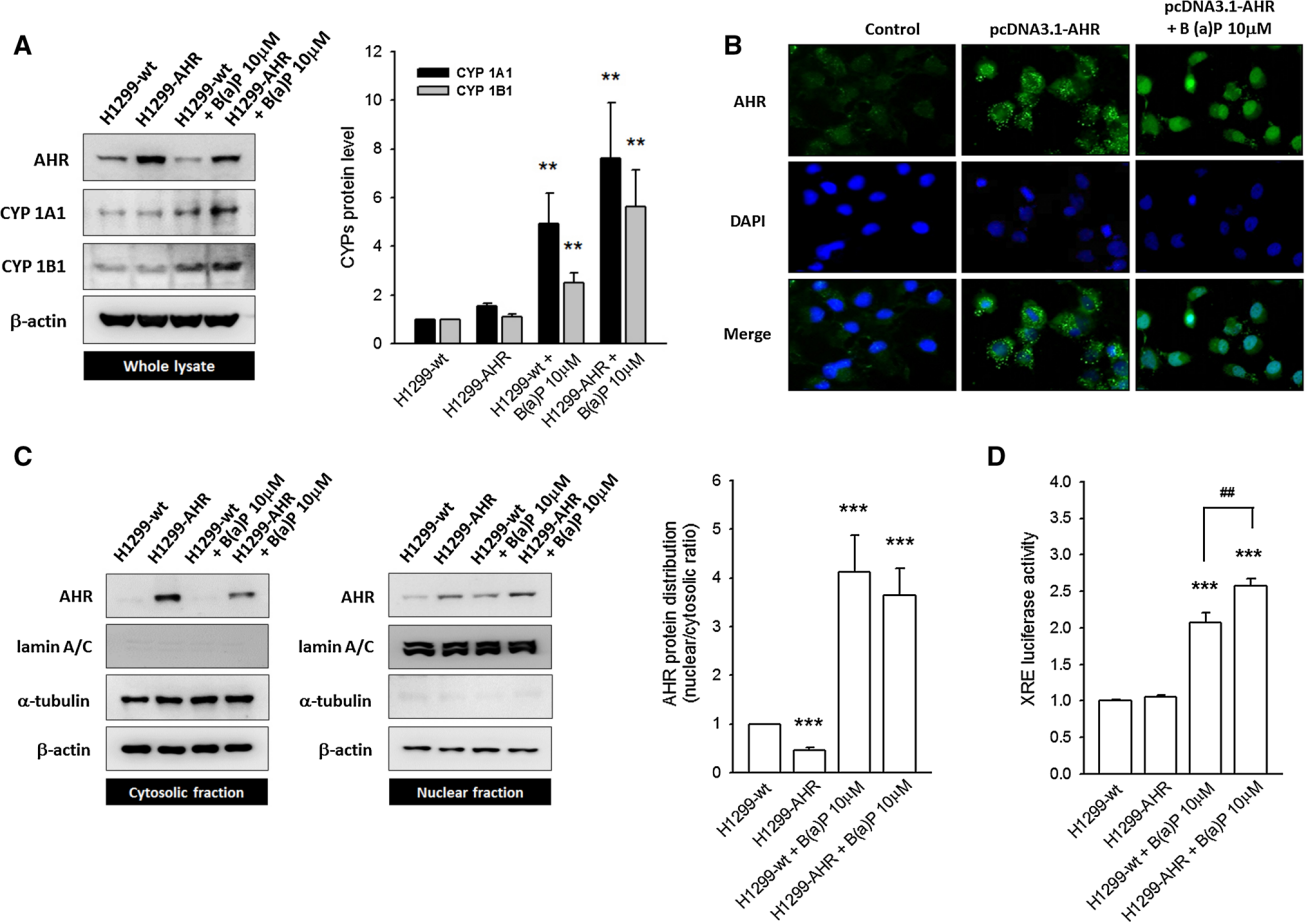
The overexpressed AHR protein predominantly localizes in the cytosol, rather than the nucleus. **a** Representative images and quantitative data illustrate the induction of CYP1A1 and CYP1B1 after B(a)P (10 μM) treatment. In H1299-AHR, neither CYP1A1 nor CYP1B1 is induced. **b** Immunofluorescent staining shows the overexpressed AHR protein located mainly in the cytoplasm. In the absence of B(a)P treatment, no AHR nuclear translocation occurs. **c** The distribution of AHR was determined using nuclear fractionation. Quantitative data are presented in Supplementary Fig. 5. B(a)P treatment causes a pronounced AHR N/C ratio, in comparison with non-treated groups. **d** The transactivation of XRE reporter is detected in B(a)P-treated groups (H1299-wt and H1299-AHR). In the absence of B(a)P stimulation, the overexpressed AHR protein alone did not induce XRE reporter activities. **p < 0.01 and ***p < 0.001 indicate a statistically significant difference from H1299-wt; ##*p* < 0.01 indicates a statistically significant difference from H1299-wt treated with B(a)P

The binding of B(a)P, a well-known exogenous AHR ligand, results in the rapid translocation of AHR into the nucleus where it functions as a transcriptional regulator and induces CYP1A1 and CYP1B1 expression. Accordingly, an increase in CYP1A1 and CYP1B1 protein levels is observed in both H1299-wt (4.93 ± 1.26- and 2.50 ± 0.4-fold increase, respectively) and H1299-AHR cells (7.59 ± 2.31- and 5.61 ± 1.53-fold increase, respectively) treated with 10 µM B(a)P as compared to untreated cells (Fig. 4a). To determine the subcellular distribution of overexpressed AHR, both immunofluorescent staining and nuclear fractionation methods were used. The FITC-stained AHR is located mainly in the cytosol of both H1299-wt and H1299-AHR cells in the absence of B(a)P; upon incubation with B(a)P, AHR translocates to the nucleus (Fig. 4b). This pattern of AHR localization was recapitulated in fractionation studies wherein B(a)P treatment of H1299-wt or H1299-AHR reduces AHR levels in the cytosolic fraction and causes a concomitant increase in AHR levels in the nuclear fraction. This increase in nuclear AHR levels is further enhanced in B(a)P-treated H1299-AHR (Fig. 4c; supplementary Fig. 5). In comparison with groups without B(a)P treatment, B(a)P elicited a pronounced AHR N/C ratio (nuclear/cytosol AHR). Moreover, this ratio correlated with the induction of CYPs as well as XRE reporter activities (Fig. 4d). These data demonstrate that overexpressed and unliganded AHR predominantly resides in the cytoplasm, where it may bear a novel, tumor suppressor-like function and prevent EMT.

### AHR–vimentin protein complex is formed in the cytoplasm resulting in proteasome degradation of vimentin

**Fig. 5:**
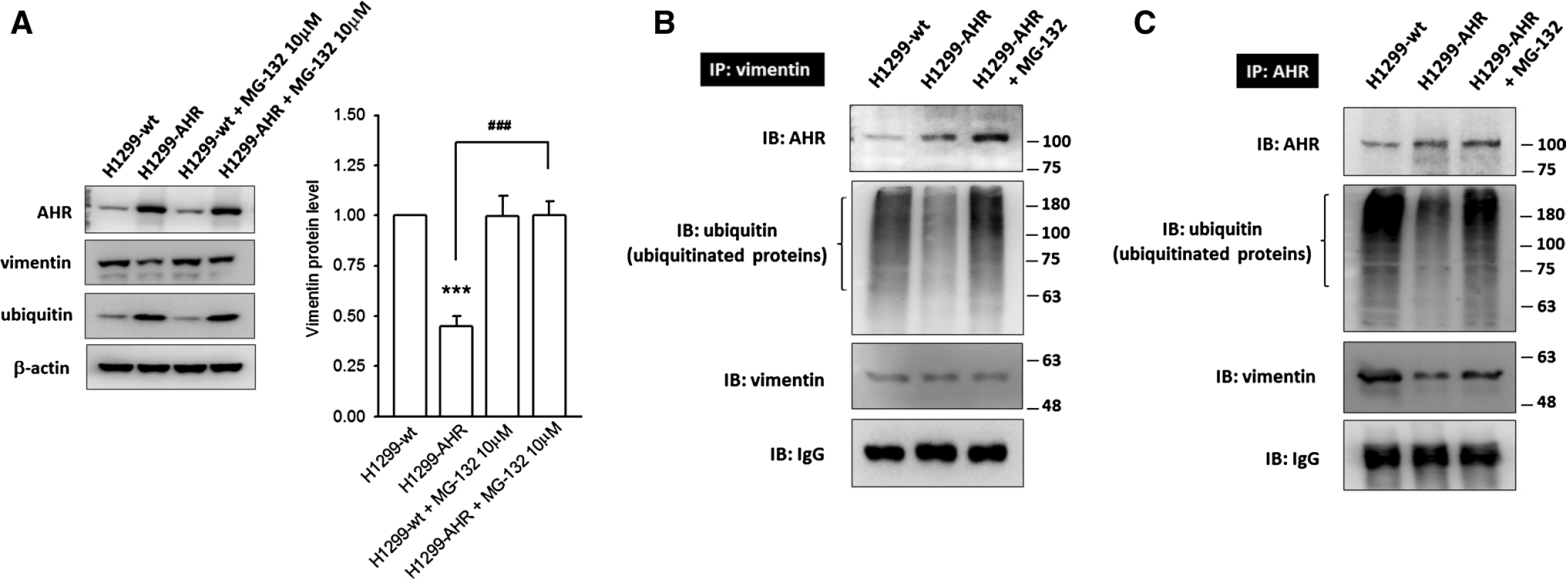
AHR–vimentin protein complex is formed in the cytoplasm and results in proteasome degradation of vimentin. **a** Representative images and quantitative data show that AHR-mediated vimentin degradation can be rescued by MG-132 (26S proteasome inhibitor) treatment. In addition, monomeric ubiquitin levels increase in H1299-AHR. ***p < 0.001 indicates a statistically significant difference from H1299-wt; ###*p* < 0.001 indicates a statistically significant difference from H1299-AHR. **b**/**c** Co-immunoprecipitation studies demonstrate the formation of AHR–vimentin protein complex in cytoplasm. IgG is used as loading control. In H1299-AHR group, an inferior signal of ubiquitinated proteins is detected, whereas this signal is enhanced in H1299-AHR group treated with MG-132, suggesting that the AHR–vimentin interacting complex might facilitate vimentin poly-ubiquitination and vimentin proteasome degradation. However, in H1299-wt group, although the AHR-expression level is weak, the small amount of AHR still shows a strong interaction with vimentin, as well as poly-ubiquitinated proteins, which suggests a frequent renewal of vimentin

We observed that the reduction in vimentin protein expression in the presence of excess cytoplasmic AHR was accompanied by an increase in monomeric ubiquitin levels, and therefore wanted to test whether the proteasome pathway was involved in this process. Treatment with MG-132 (10 μM; 26S proteasome inhibitor) for 6 h fully restored the vimentin level in H1299-AHR, suggesting the involvement of proteasome pathway in AHR-mediated vimentin degradation (Fig. 5a). AHR was previously identified as an E3 ubiquitin ligase (Ohtake et al. 2007, 2009). In this study, the formation of AHR–vimentin interacting complex was demonstrated by co-immunoprecipitation (Fig. 5b, c). In H1299-AHR cells, a fraction of AHR–vimentin protein complex with poor poly-ubiquitination was captured using an antivimentin or anti-AHR antibody. MG-132 treatment not only increases the amount of captured AHR–vimentin complex, but also causes the accumulation of ubiquitinated proteins, suggesting that AHR interaction with vimentin in the cytoplasm might facilitate vimentin ubiquitination, leading to proteasome-mediated degradation. On the other hand, in H1299-wt cells that poorly express AHR, it is intriguing to note that a small amount of AHR is capable of interacting strongly with vimentin and ubiquitinated proteins, especially when anti-AHR capture antibody was used. These results suggest that a high turnover of vimentin takes place in H1299-wt to maintain its invasive potential.

### AHR-mediated vimentin degradation and EMT suppression are reversed when glycogen synthase kinase 3 beta (GSK3β) is inactivated

**Fig. 6:**
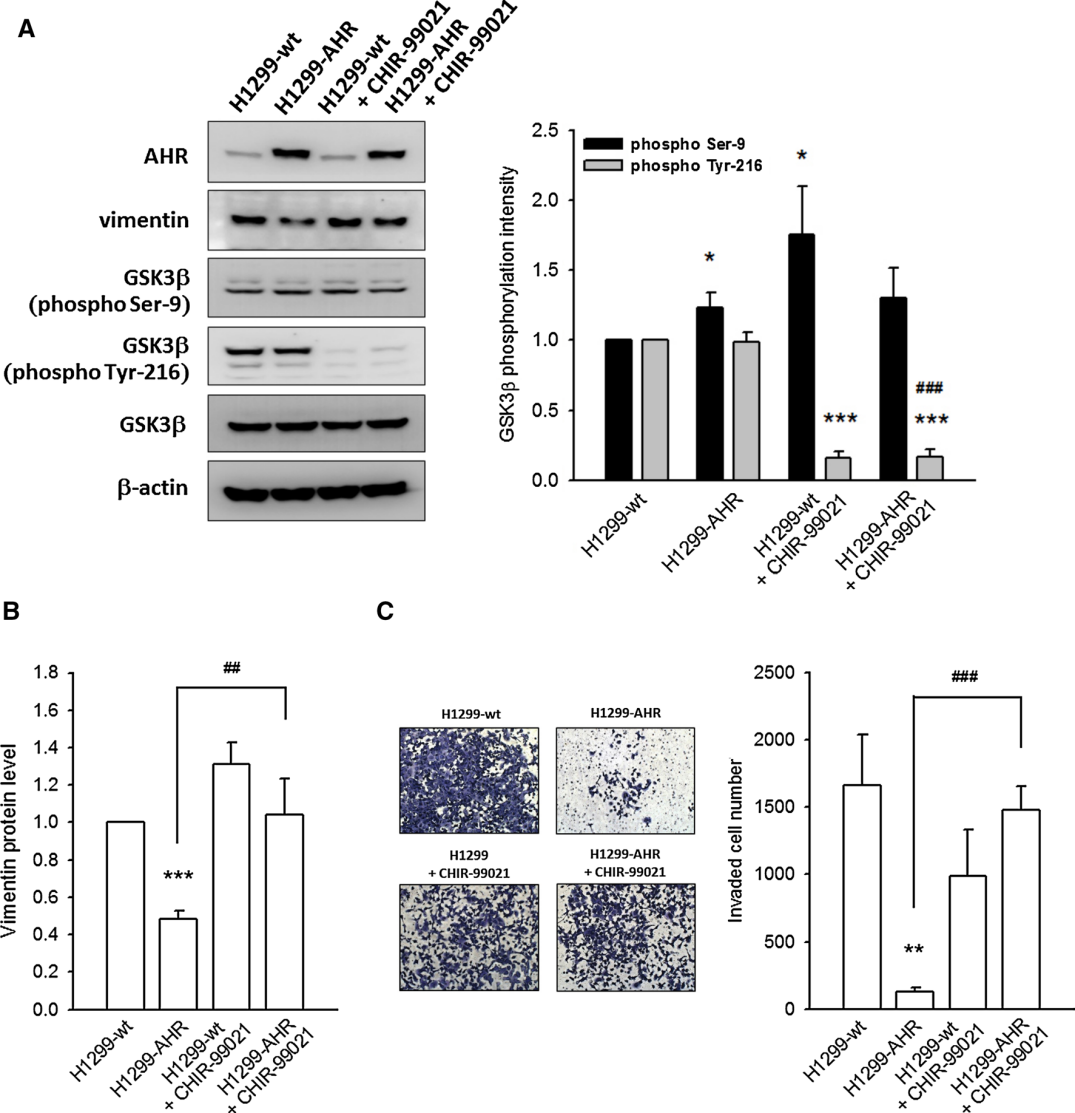
AHR-mediated vimentin degradation and EMT inhibition are reversed when GSK3β is inactivated. **a** Representative images and quantitative data show the intensity of GSK3β phosphorylation on Ser-9 or Tyr-216 residues. CHIR-99021 (GSK3β inhibitor, 2 μM) treatment of H1299-AHR for 6 h completely inhibits Tyr-216 phosphorylation of GSK3β. **b** CHIR-99021 treatment rescues AHR-mediated vimentin degradation. **c** Representative images and quantitative data show CHIR-99021 treatment reverses AHR-mediated invasion prevention. *p < 0.05, **p < 0.01, and ***p < 0.001 indicate a statistically significant difference from the control group (H1299-wt); ##*p* < 0.01 and ###*p* < 0.001 indicate a statistically significant difference from H1299-AHR

In malignant cancers, glycogen synthase kinase 3 beta (GSK3β) is inactivated, and this inactivation is known to promote cell migration and invasion. In H1299-AHR cells, a slight increase in GSK3β Ser-9 phosphorylation (represents inactive GSK3β) is observed, whereas the GSK3β Tyr-216 phosphorylation (represents active GSK3β), GSK3β protein level, and the GSK3β inactivation index (phospho-Ser-9/phospho-Tyr-216 ratio) are significantly unchanged, as compared to H1299-wt (Fig. 6a; supplementary Fig. 7A). Treatment with CHIR-99021 (2 μM, GSK3β inhibitor) for 6 h not only inhibited Tyr-216 phosphorylation completely (Fig. 6a), but also repressed AHR-mediated vimentin degradation (Fig. 6b) and invasion prevention (Fig. 6c).

### Silencing of AHR expression leads to EMT in A549 cells

**Fig. 7:**
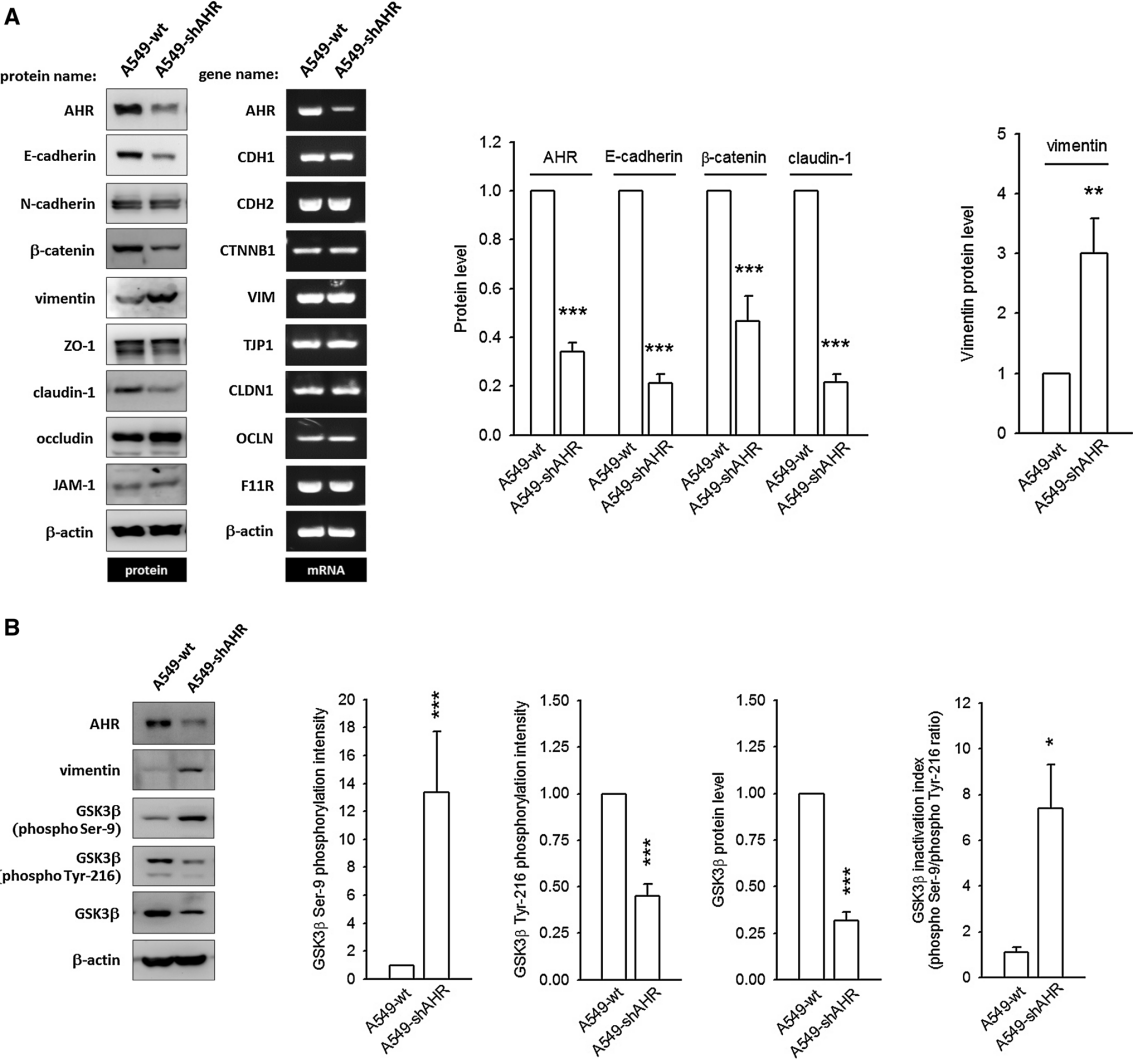
Malignant characteristics develop in AHR-silenced A549 cells. A549-shAHR (AHR-silenced A549) was established as described in Materials and Methods.** a** Representative images of immunoblot and agarose gel electrophoresis of PCR products to detect and compare protein and mRNA expression of epithelial–mesenchymal biomarkers between A549-wt and A549-shAHR. None of the detected genes is altered at the mRNA level, with the exception of AHR, which was silenced by shRNA. However, the protein expression levels of E-cadherin, β-catenin, and claudin-1 are markedly reduced, whereas vimentin expression increases.** b** AHR knock-down causes a strong augmentation in GSK3β Ser-9 phosphorylation, and an alleviation in GSK3β Tyr-216 phosphorylation, which results in an overt GSK3β inactivation index. The GSK3β protein level is reduced in A549-shAHR. These data demonstrate that the cytoplasm-residing AHR functions as a guard and prevents the expression of mesenchymal factors. **p < 0.01 and ***p < 0.001 indicate a statistically significant difference from the control group

A549-wt cells usually exhibit a pebble-like morphology and strong intercellular adhesion. However, in A549-shAHR cells (AHR-silenced A549), cell–cell contacts are degenerated and cells adopt a more elongated shape (supplementary Fig. 8A), which is reminiscent of the morphology of cells treated with TGF-β1 (an EMT inducer). In an in vitro invasion assay, a higher number of A549-shAHR-invaded cells are obtained (supplementary Fig. 8B), suggesting that AHR silencing might lead to the loss of tumor suppressor-like function and hence promote EMT. The epithelial–mesenchymal signatures of A549-wt and A549-shAHR are characterized in Fig. 7a. When AHR expression is knocked-down, A549-wt loses its epithelial characteristics, as evidenced by an obvious reduction in E-cadherin, β-catenin, and claudin-1 protein levels and an increase (3.0 ± 0.58-fold) in mesenchymal vimentin expression. This pattern is similar to the one seen in AHR-deficient H1299 cells (Fig. 1). However, the mRNA levels of these biomarkers are unaltered. Interestingly, AHR silencing strongly augments GSK3β Ser-9 phosphorylation (13.4 ± 4.4-fold increase) and simultaneously alleviates GSK3β Tyr-216 phosphorylation, resulting in an overt GSK3β inactivation index (phospho-Ser-9/phospho-Tyr-216 ratio). The expression of GSK3β protein is also reduced. Taken together, these data clearly demonstrate that the AHR protein resident in the cytoplasm can prevent the expression of mesenchymal markers and downregulate cancer cell metastasis.

## Discussion

In mammals, AHR is almost ubiquitously expressed among organs, but its expression levels vary widely. The highest levels are detected in the liver, lungs, and kidneys, as well as most of the cells of epithelial origin. Therefore, we can speculate AHR possesses physiological functions besides metabolism. AHR-null mice phenotypically exhibit developmental and functional deficits in several tissues (Harstad et al. 2006; Singh et al. 2011; Stevens et al. 2009). Accumulating in vitro studies also provide substantial support for AHR involvement in cell proliferation (Abdelrahim et al. 2003), apoptosis (Fan et al. 2010), cell migration (Carvajal-Gonzalez et al. 2009b), immune regulation (Navid et al. 2013), and cancer progression (Safe et al. 2013).

AHR-expression levels, at least in some tumors, are correlated with poor differentiation and prognosis clinically. However, in other tumors, an inverse correlation is found, which suggests that the role of AHR during progression of different tumors is complicated (Portal-Nuñez et al. 2012). Generally, cytoplasmic AHR and nuclear AHR are predominantly observed in non-neoplastic tissues and in malignant tissues, respectively (Ishida et al. 2015). Ligand binding alters AHR conformation to expose its nuclear localization sequence (NLS), which results in nuclear import. After that, the activated AHR is eliminated either by nuclear exportation and ubiquitin–proteasome degradation or by its negative feedback repressor (Ikuta et al. 2009; Stevens et al. 2009). Thus, the accumulation of AHR in the nuclei of malignant tumors can be attributed to the following abnormalities: the aberrant activation of transcriptional regulators [such as protein kinase CK2, signal transducer, and activator of transcription 6 (STAT6) and NFκB] that enhance AHR expression (Belguise et al. 2007); obstructions in the negative feedback loop; and most importantly, constitutive AHR activation (even in the absence of ligands). Expression of constitutively active AHR in vivo heightens the susceptibility to neoplastic lesions (Andersson et al. 2002; Moennikes et al. 2004) and facilitates the differentiation of Treg cells (Navid et al. 2013). These data demonstrate that nuclear AHR is a potent oncogenic factor. In this study, we corroborated that NSCLC cells with abundant AHR in the cytoplasm (e.g., A549, H1299-AHR, CL1-0) are poorly invasive, whereas invasive potential is enhanced in AHR-deficient cells (such as A549-shAHR, H1299-wt, CL1-5). Similar results were also obtained in neuroblastoma, where ectopic expression of AHR was found to downregulate MYCN (specific malignant factor of neuroblastoma) and E2F1 expression and correlated highly with the histological grade of differentiation. Induction of MYCN and E2F1 expression was observed when AHR is silenced (Wu et al. 2014). Collectively, these data prove that AHR-expression level is inversely correlated with malignancy, especially EMT, in various cancer cells.

In many types of cancer, the existence of AHR agonists has been demonstrated that can augment the expression of inflammatory mediators (Miller et al. 2005), MMPs (Peng et al. 2009a; Ishida et al. 2010), and transcriptional regulators (Pierre et al. 2014) in a genomic pathway, which leads to downregulation of cell adhesion and upregulation of migration and invasion. However, in this study, the repression of invasiveness in H1299-AHR was a result of a decrease in vimentin content. In contrast, the amount of vimentin increased markedly in A549-shAHR cells, thereby increasing the invasive potential of these cells. A reduction in the expression of E-cadherin, β-catenin, and claudin-1 also contributed to EMT in A549-shAHR. Importantly, these changes were limited to the protein level, while mRNA content was unchanged. In H1299-AHR, vimentin degradation could be abrogated by proteasome inhibition, which suggests that unliganded AHR residing in the cytoplasm can accelerate vimentin degradation in a non-genomic manner. In addition to its role as a ligand-activated transcription factor, AHR has also been identified as a novel E3 ubiquitin ligase, which facilitates the assembling of ubiquitin ligase complex and accelerates proteolysis of certain nuclear receptors (Ohtake et al. 2007, 2009). AHR has been found to suppress intestinal carcinogenesis through E3 ubiquitin ligase-dependent β-catenin degradation (Kawajiri et al. 2009). In this study, by using co-immunoprecipitation assay, we found that cytoplasmic AHR could interact with vimentin, followed by an increase in poly-ubiquitination, eventually leading to protein degradation.

The activation of AHR via a non-genomic pathway has been attracting interest in EMT regulation. For example, phthalates-stimulated AHR couples with G proteins and triggers the PI-3 kinase/Akt/NF-κB and ERK/VEGF cascades, which contributes to metastasis and angiogenesis, respectively (Tsai et al. 2014). The Src kinase, an important factor in maintenance of cadherin-dependent intercellular contact, is also known to be associated with AHR complex. Hexachlorobenzene treatment immediately dissociates Src kinase from AHR, thereby activating its own kinase activity and destabilizing cell adhesion (Randi et al. 2008). In cultured fibroblasts derived from AHR^−/−^ mice, an enhanced spreading and adhesion correlate with decreased Vav-3 and Rac1 activities and increased RhoA/Rho kinase (Rock) pathway activity (Carvajal-Gonzalez et al. 2009a, b). These findings demonstrate that multiple signaling factors can be regulated by AHR through a non-genomic pathway. In this work, we found that cytoplasmic AHR can regulate GSK3β signaling. GSK3β activation usually induces phosphorylation of its substrates (e.g., β-catenin and cyclin D1), thus promoting ubiquitination and proteasome degradation of these substrates. The inactivation of GSK3β has been identified as an important event in cancer progression (Mishra et al. 2015). In this study, pronounced phosphorylation and de-phosphorylation of GSK3β Ser-9 and Tyr-216 residues, respectively, were observed in A549-shAHR cells, suggesting that the loss of AHR predisposes to GSK3β inactivation. Endogenous GSK3 activity is required for maintenance of the integrity of apical junction complexes, whereas the reduction of GSK3 activity will result in decreased protein level of claudin-1 and E-cadherin, but without influencing on ZO-1 and JAM-1 (Severson et al. 2010), which is similar to what we found in A549-shAHR. Although AHR overexpression has no effect on GSK3β inactivation index in H1299-AHR, treatment with GSK3β inhibitor did prevent vimentin degradation and restored invasive potential. These data suggest that the relative level of AHR in the cytoplasm is crucial for the maintenance of GSK3β in active form, as the downregulation of AHR expression results in GSK3β inactivation and EMT.

The role of AHR in tumor pathology is complicated and not fully understood yet. In this work, we showed that an inverse relationship exists between AHR protein levels and the invasive ability of NSCLC cells. We have demonstrated for the first time that the cytoplasm-residing, unliganded AHR protein functions as a guard and prevents the expression of mesenchymal factors. Through its E3 ligase activity, AHR maintains GSK3β in an active form and accelerates vimentin degradation, thus suppressing invasive behavior and EMT via a non-genomic pathway. Although AHR in cytoplasm possesses tumor suppressor-like function, unfortunately, it is also likely to act as a potent inducer of malignancy. The presence of AHR agonists will trigger AHR translocation into the nucleus, where it can activate a genomic pathway favoring tumor survival and progression (supplementary Fig. 9). Thus, AHR may be an attractive target with dual effects during cancer treatment.

## Electronic supplementary material

Below is the link to the electronic supplementary material.

Supplementary material 1 (PDF 796 kb)

## Abbreviations

AHR: Aryl hydrocarbon receptor
EMT: Epithelial–mesenchymal transition
GSK3β: Glycogen synthase kinase 3 beta
NSCLC: Non-small cell lung cancer
JAM-1: Junction adhesion molecule-1
ZO-1: Zonula occludens-1
TCDD: 2,3,7,8-Tetrachlorodibenzo-p-dioxin
B(a)P: Benzo(a)pyrene
CYP1A1: Cytochrome P450 1A1
MMP: Matrix metalloproteinase
