# Supplementary material for: Cytoplasmic aryl hydrocarbon receptor regulates glycogen synthase kinase 3 beta, accelerates vimentin degradation, and suppresses epithelial–mesenchymal transition in non-small cell lung cancer cells
Source: Arch Toxicol. 2016 Oct 17;91(5):2165–78. doi: 10.1007/s00204-016-1870-0 (PMC5399057; doi:10.1007/s00204-016-1870-0)
Supplement: Supplementary file 1 — Supplementary material 1 (PDF 796 kb) [file 204_2016_1870_MOESM1_ESM.pdf]

SUPPLEMENTARY INFORMATION

Supplementary Table 1:

The Primer Sets of Genes (and Encoded Protein Information) Presented in This Study

HGNC Official Name (Official Symbol) [#1]	Accession No. (Gene ID) [#2]	Protein information Preferred Names	Sense / Antisense sequences [Product size (b.p.)]
aryl hydrocarbon receptor (AHR)	NM_001621 (196)	aryl hydrocarbon receptor (AHR)	CAGCCTCAGGATGTGAACTC / TTACAGGAATCCACTGGATGT [1002]
cadherin 1 (CDH1)	NM_004360 (999)	CD324, cadherin-1, E-cadherin (epithelial)	TGGAGGAATTCTTGCTTTGC / CGTACATGTCAGCCAGCTTC [488]
cadherin 2 (CDH2)	NM_001792 (1000)	CD325, cadherin-2, N-cadherin (neuronal)	CACTGCTCAGGACCCAGAT / TAAGCCGAGTGATGGTCC [416]
catenin beta 1 (CTNNB1)	NM_001904 (1499)	beta-catenin, catenin beta-1	TTGATGGAGTTGGACATGG / CAGGACTTGGGAGGTATCCA [171]
Vimentin (VIM)	NM_003380 (7431)	vimentin	GAGAACTTTGCCGTTGAAGC / GCTTCCTGTAGGTGGCAATC [163]
tight junction protein 1 (TJP1)	NM_003257 (7082)	zona occludens 1 (ZO-1), tight junction protein ZO-1	CACCTTTTGATAATCAGCACTCTCA / CTCTAGGTGCCTGTTCGTAACGT [144]
claudin 1 (CLDN1)	NM_021101 (9076)	claudin-1, senescence-associated epithelial membrane protein 1	CCTATGACCCCAGTCAATGC / TCACACGTAGTCTTTCCC [186]
Occludin (OCLN)	NM_002538 (100506658)	occludin, tight junction protein occludin	TCAGGGAATATCCACCTATCACTTC AG / CATCAGCAGCAGCCATGTACTCTTC AC [189]
F11 receptor (F11R)	NM_016946 (50848)	CD321, junctional adhesion molecule A (JAM-A), junctional adhesion molecule 1 (JAM-1), platelet F11 receptor	GGTCAAGGTCAAGCTCAT / CTGAGTAAGGCAAATGCAG [582]
actin beta (ACTB)	NM_001101 (60)	actin beta	TCATGAGGTAGTCAGTCAGG / TGACCCAGATCATGTTTGAG [215]

[#1] The official human gene names/symbols are approved by HUGO Gene Nomenclature Committee (http://www.genenames.org/about/guidelines).

[#2] Accession number identified the sequence record for primer design is referred from NCBI GenBank.

Supplementary Figure 1.

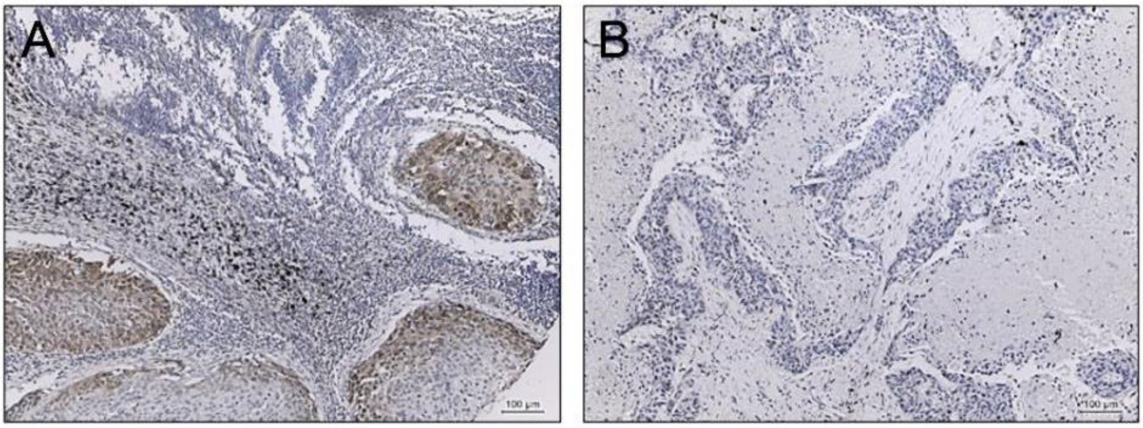

Supplementary Figure 1. AHR down-regulation is correlated with the malignancy of human lung cancer tissues. Representative images of IHC staining of AHR in specimens from 30 lung cancer patients. The expression level of AHR was obvious in low-grade (A) but not high-grade (B) tumors.

Supplementary Figure 2.

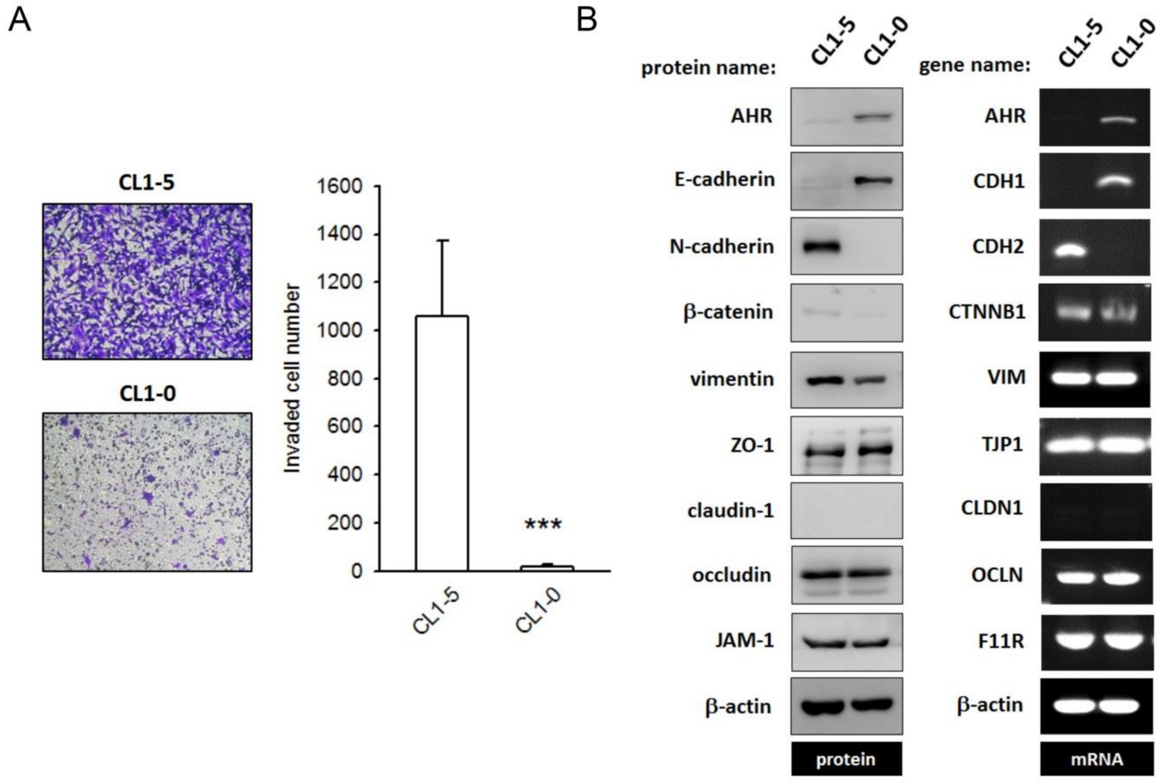

Supplementary Figure 2. The invasion potential of CL1-0 and CL1-5 is inversely related to their amount of AHR protein expressed. (A) Both representative images and quantitative data demonstrated CL1-5 is more aggressive than CL1-0, by using Matrigel pre-coated transwell invasion assay. ***$p < 0.001$ indicates a statistically significant difference from CL1-5. (B) The protein and mRNA expression levels of epithelial/mesenchymal biomarkers were detected and compared between CL1-0 and CL1-5. An inverse relationship was found between AHR protein expression level and vimentin. For HGNC name/symbol of genes and encoded proteins, please refer to Supplementary Table 1.

Supplementary Figure 3.

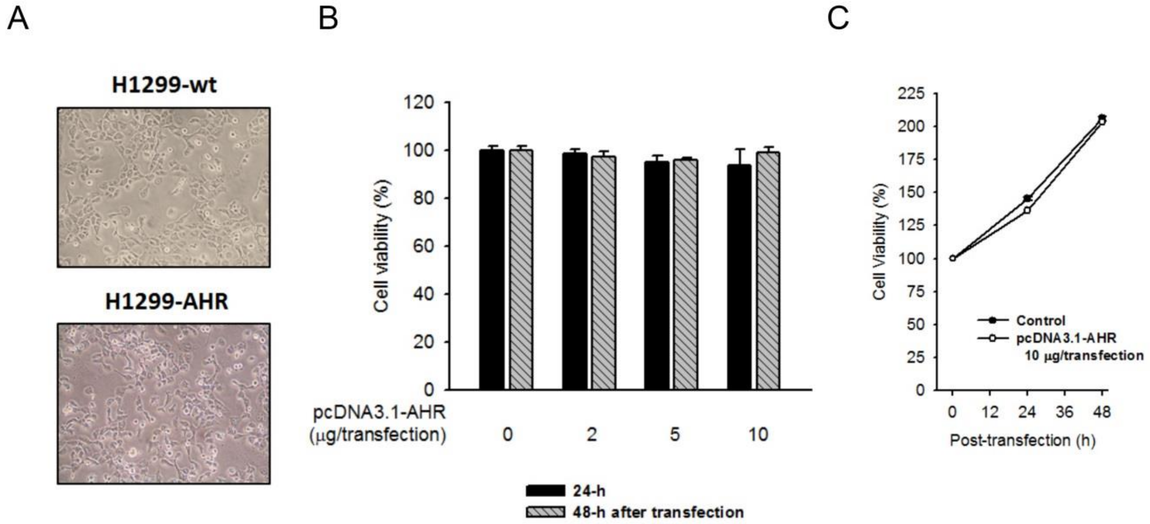

Supplementary Figure 3. The cell morphology and proliferation curve of H1299-AHR. (A) As shown in phase-contrast images, no obvious changes in cell morphology were found in H1299-AHR, as compared to H1299-wt. (B/C) No differences in cytotoxicity (B) and proliferative curve (C) were observed between H1299-wt and H1299-AHR.

Supplementary Figure 4.

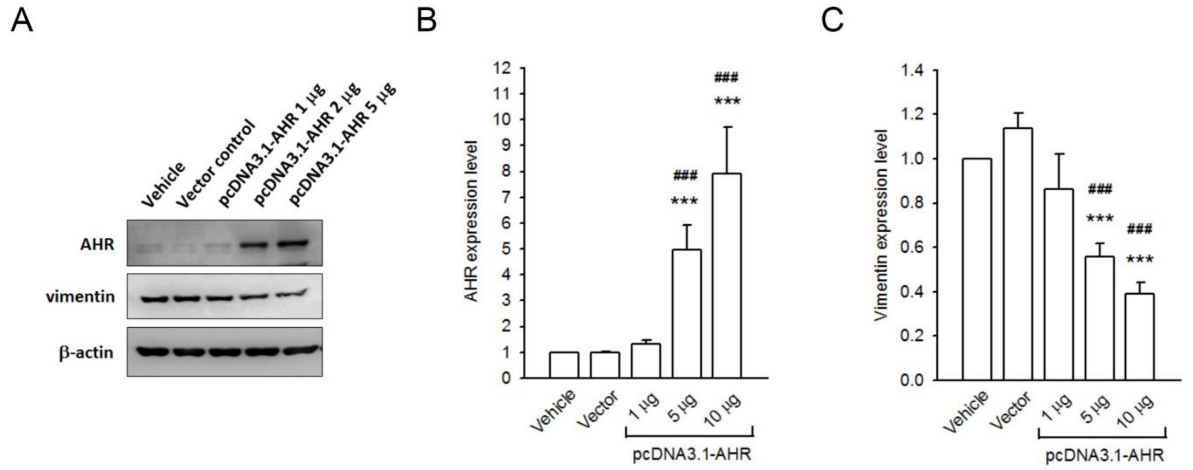

Supplementary Figure 4. The expression of mesenchymal vimentin is reduced in H1299-AHR. Both representative images (A) and quantitative data (B/C) demonstrated AHR was constitutively expressed in H1299 after pcDNA3.1-AHR transfection. The amount of AHR protein expressed was correlated with the amount of plasmid transfected. The expression level of vimentin was decreased, and showed an inverse correlation to the amount of AHR expressed. Vector control has no effects on AHR and vimentin expression levels. ***$p < 0.001$ indicates a statistically significant difference from control (H1299-wt). ###$p < 0.001$ indicates a statistically significant difference from vector control.

Supplementary Figure 5.

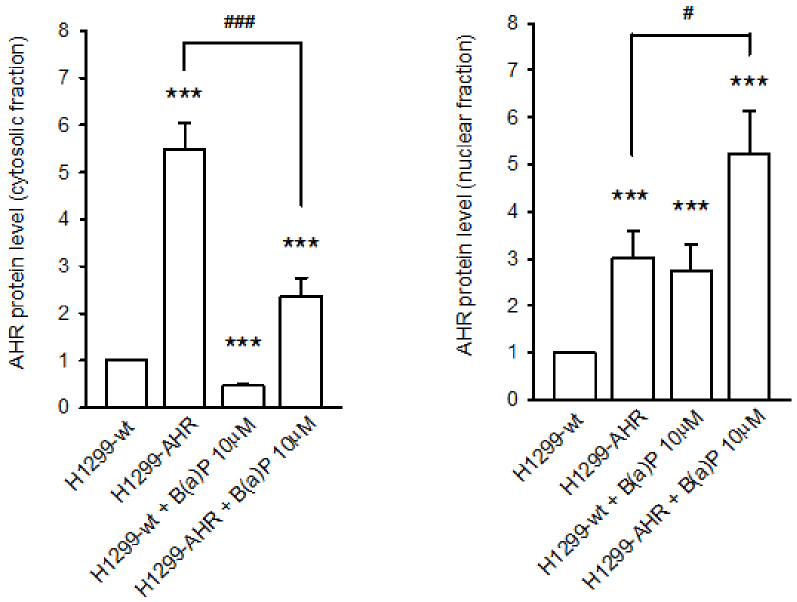

Supplementary Figure 5. AHR distribution in H1299-wt (or H1299-AHR) treated with B(a)P or not. After 6 h incubation with 10 μM B(a)P, the cytosolic fraction and nuclear fraction were isolated as described in Material and Methods. AHR distribution was examined by Western blots, and the representative blots were presented in Figure 4. Quantitative data showed the distribution of AHR protein in cytosolic fraction (left panel) and nuclear fraction (right panel). Although an accumulation of AHR was found in nucleus of H1299-AHR, however, this might be an artificial effect (because no CYPs was induced and without influences on XRE reporter). ***$p < 0.001$ indicates a statistically significant difference from H1299-wt. #$p < 0.05$, and ###$p < 0.001$ indicates a statistically significant difference from H1299-AHR.

Supplementary Figure 6.

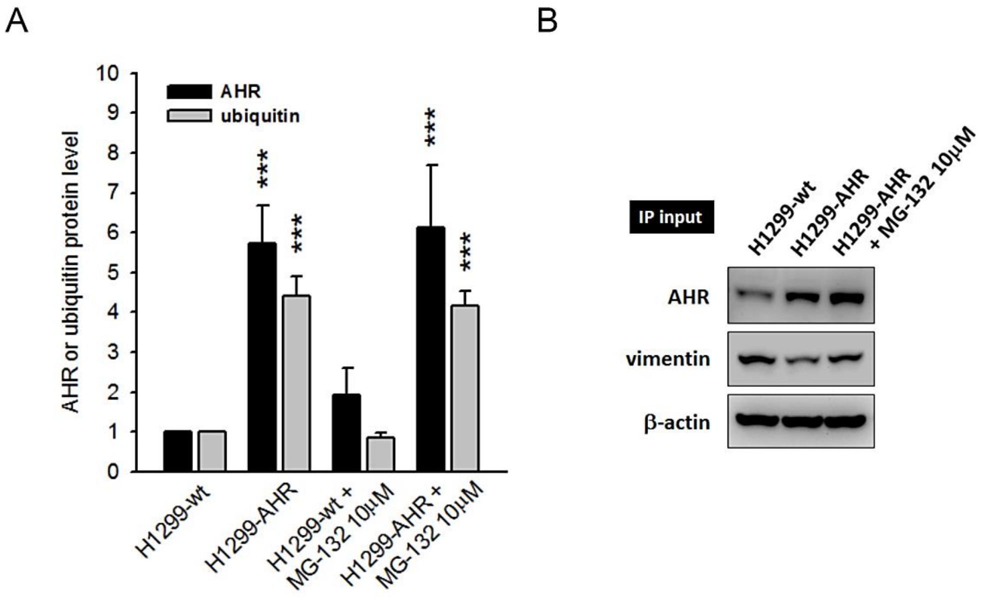

Supplementary Figure 6. AHR-vimentin protein complex is formed in the cytoplasm, and results in proteasome degradation of vimentin. (A) An extra-quantitative data of Figure 5A. The presence of excess cytoplasmic AHR was accompanied by an increase in monomeric ubiquitin levels. MG-132 treatment did not change AHR and monomeric ubiquitin level. ***$p < 0.001$ indicates a statistically significant difference from H1299-wt. (B) The IP input of Figure 5B/5C.

Supplementary Figure 7.

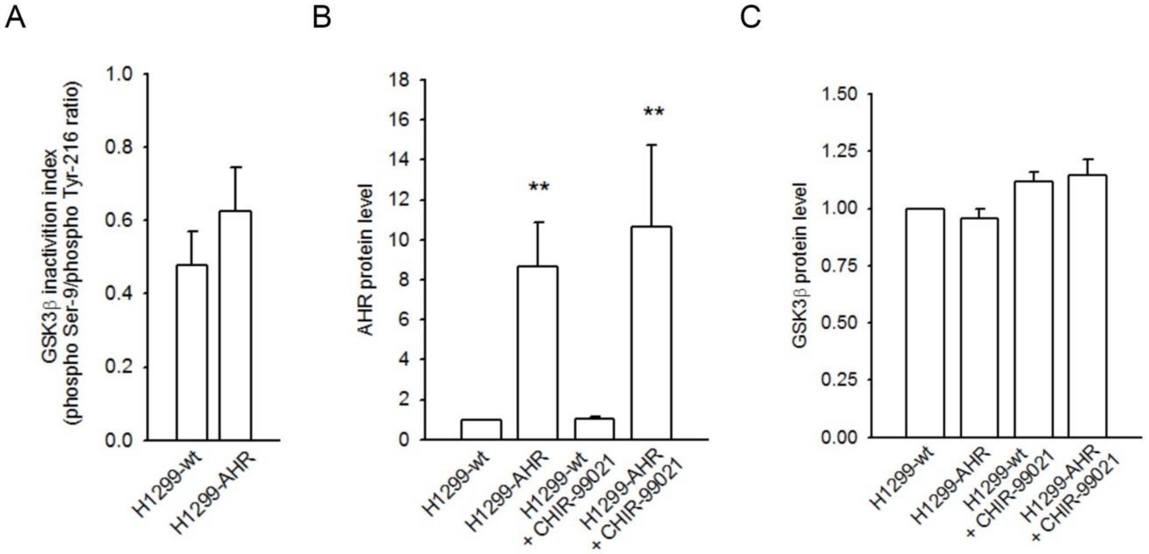

Supplementary Figure 7. AHR-mediated vimentin degradation and EMT suppression is reversed when GSK3β is inactivated (extra-quantitative data of Figure 6). (A) GSK3β inactivation index, which expressed as "phospho-Ser-9/phospho-Tyr-216 ratio", shows no differences between H1299-wt and H1299-AHR. (B/C) CHIR-99021 treatment did not change AHR and GSK3β protein level. **$p < 0.01$ indicates a statistically significant difference from the control group.

Supplementary Figure 8.

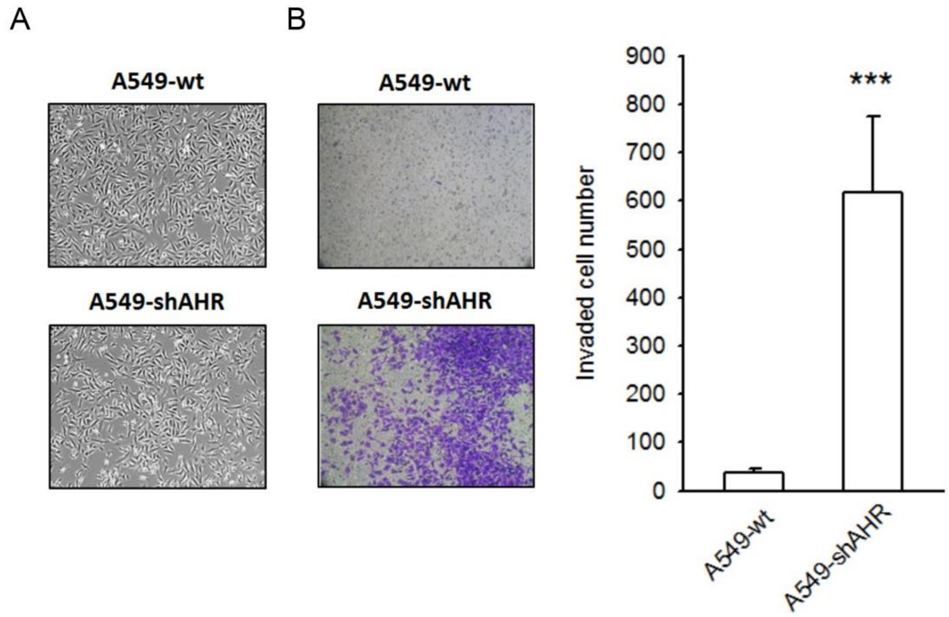

Supplementary Figure 8. Malignant characteristics develop in AHR-silenced A549 cells. (A) The pebble-like morphology and strong intercellular adhesion of A549-wt was taken in photograph. But, A549-shAHR showed an elongated, fibroblast-like shape and degeneration among cell contacts. (B) Both representative images and quantitative data illustrated a pronounced enhancement of invasiveness potential in A549-shAHR, as compared to A549-wt. ***p < 0.001 indicates a statistically significant difference from the control group.

Supplementary Figure 9.

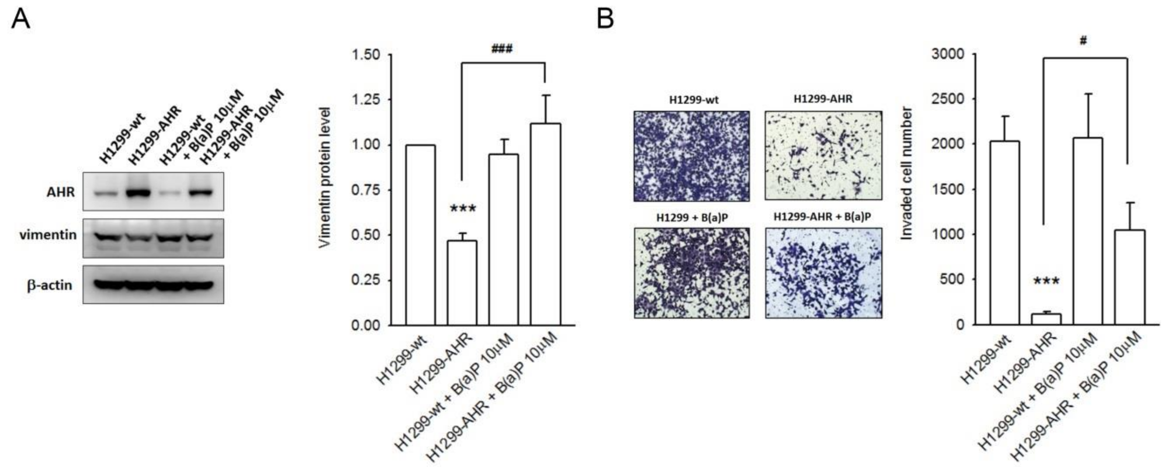

Supplementary Figure 9. Cytoplasmic AHR mediated vimentin degradation and EMT inhibition is reversed when AHR activation. (A) B(a)P (AHR agonist, 10 μM) treatment of H1299-AhR for 24 h, completely rescues AHR-mediated vimentin degradation. (B) B(a)P treatment reverses AHR-mediated invasion prevention. ***$p < 0.001$ indicates a statistically significant difference from the control group (H1299-wt); # $p < 0.05$ and ### $p < 0.001$ indicates a statistically significant difference from H1299-AHR.
